# Validation of the standardised assessment of personality – abbreviated scale in a general population sample

**DOI:** 10.1002/pmh.1307

**Published:** 2015-08-27

**Authors:** Marcella Lei‐Yee Fok, Seth Seegobin, Souci Frissa, Stephani L. Hatch, Matthew Hotopf, Richard D. Hayes, Paul Moran

**Affiliations:** ^1^ Department of Health Service and Population Research, Institute of Psychiatry, Psychology & NeuroscienceKing's College LondonLondonUK; ^2^ MRC Social, Genetic and Developmental Psychiatry Centre, Institute of Psychiatry, Psychology & NeuroscienceKing's College LondonLondonUK; ^3^ Department of Psychological Medicine, Institute of Psychiatry, Psychology & NeuroscienceKing's College LondonLondonUK

## Abstract

**Background:**

Personality disorder (PD) is associated with important health outcomes in the general population. However, the length of diagnostic interviews poses a significant barrier to obtaining large scale, population‐based data on PD. A brief screen for the identification of people at high risk of PD in the general population could be extremely valuable for both clinicians and researchers.

**Aim:**

We set out to validate the Standardised Assessment of Personality – Abbreviated Scale (SAPAS), in a general population sample, using the Structured Clinical Interviews for DSM‐IV Personality Disorders (SCID‐II) as a gold standard.

**Method:**

One hundred and ten randomly selected, community‐dwelling adults were administered the SAPAS screening interview. The SCID‐II was subsequently administered by a clinical interviewer blind to the initial SAPAS score. Receiver operating characteristic analysis was used to assess the discriminatory performance of the SAPAS, relative to the SCID‐II.

**Results:**

Area under the curve for the SAPAS was 0.70 (95% CI = 0.60 to 0.80; *p* < 0.001), indicating moderate overall discriminatory accuracy. A cut point score of 4 on the SAPAS correctly classified 58% of participants. At this cut point, the sensitivity and specificity were 0.69 and 0.53 respectively.

**Conclusion:**

The SAPAS operates less efficiently as a screen in general population samples and is probably most usefully applied in clinical populations. © 2015 The Authors Personality and Mental Health published by John Wiley & Sons Ltd

## Introduction

Personality disorder (PD) is a global health problem (Tyrer et al., 2010). It is one of the hardest psychiatric conditions to treat and is associated with substantial morbidity and significantly raised mortality from both natural and unnatural causes (Fok, Stewart, Hayes, & Moran, 2014b; Fok et al., 2012).

At the level of the community, PD is a prevalent mental disorder associated with considerable public health burden. In the World Health Organization World Mental Health Surveys of DSM‐IV Personality Disorder, approximately 6% of community participants from 13 high‐income, middle‐income and low‐income countries met the criteria for a PD (Huang et al., 2009), although other surveys have found higher prevalence figures of the order of 10–13% (Samuels, 2011; Torgersen, Kringlen, & Cramer, 2001). In community settings, people with PD are more likely to be separated or divorced, unemployed, living in urban locations and are also more likely to have concurrent health problems in the form of mood, anxiety and substance use disorders (Coid, Yang, Tyrer, Roberts, & Ullrich, 2006). They also report a greater number of physical health problems and are at particularly elevated risk of cardiovascular disease (Moran et al., 2007). The assessment of PD status therefore conveys valuable information about the health status of members of the general population. However, diagnostic interviews for PD are often lengthy and require special training, and this poses a major obstacle to obtaining large‐scale, population‐based data on PD. Under such circumstances, an efficient screening interview might be usefully applied, perhaps as part of a two‐stage procedure for case identification (Lenzenweger, Loranger, Korfine, & Neff, 1997).

The Standardised Assessment of Personality – Abbreviated Scale (SAPAS) is a short and simple interview‐administered screen for PD. The SAPAS was developed from the semi‐structured interview Standardised Assessment of Personality (Mann, Jenkins, Cutting, & Cowen, 1981; Moran, Rendu, Jenkins, Tylee, & Mann, 2001) and validated in a sample of psychiatric patients, where it was found to have good psychometric properties, correctly identifying the presence of PD in 90% of patients, with a sensitivity and specificity of 0.94 and 0.85 respectively (Moran et al., 2003). Subsequent field testing of the SAPAS in samples consisting of patients with substance abuse (Gonzalez, 2014; Hesse & Moran, 2010; Hesse, Rasmussen, & Pedersen, 2008), patients with depression (Bukh, Bock, Vinberg, Gether, & Kessing, 2010; Gorwood et al., 2010), probationers (Pluck, Sirdifield, Brooker, & Moran, 2012) and incarcerated adolescent boys (Kongerslev, Moran, Bo, & Simonsen, 2012) have confirmed the validity, reliability and clinical usefulness of the instrument. The SAPAS was developed in a clinical setting, and although the instrument has been used outside secondary care (Buszewicz, Griffin, McMahon, Beecham, & King, 2010), to our knowledge, no studies have examined the screening properties of the SAPAS when applied in a general population setting. This paper reports results from a study of the performance of the SAPAS in a general population sample.

## Methods

### Participants and sample size

We selected a random sample of participants from a pool of adult participants of the South East London Community Health study (SELCOH) study (Hatch et al., 2011), a community survey of psychiatric and physical morbidity. Details of the SELCOH methodology have been described elsewhere (Hatch et al., 2011). In brief, participants were community residents in randomly selected households located within the boroughs of Southwark and Lambeth in South East London. Wave 1 of the study took place from 2008 to 2010; follow‐up (Wave 2) took place from 2011 to 2013. Ninety‐four per cent (*n* = 1596) of participants at Wave 1 agreed to be re‐contacted at Wave 2. Participants for this study were selected on the basis of their baseline SAPAS score at Wave 1. The eligible pool for recruitment into this study consisted of people who had complete SAPAS at SELCOH Wave 1 and who consented to being followed up at Wave 2 (*n* = 1565). The Structured Clinical Interview for DSM‐IV Personality Disorders SCID‐II interviewing took place at Wave 2 (see procedure in the subsequent discussion).

For the purposes of validating the SAPAS, we wanted to ensure that we had a sufficient number of ‘true cases’ of PD. We therefore oversampled screen positive cases in order to estimate the sensitivity and specificity with adequate precision. In the original validation study (Moran et al., 2003), a score of 3 or more on the SAPAS correctly identified the presence of PD in over 90% of participants (sensitivity: 0.94; specificity: 0.85). A random sample of 70 individuals with a SAPAS score of 3 or more would allow for the detection of sensitivity of 0.94 with 95% confidence intervals of 0.86–0.98; in addition, a random sample of 40 individuals with a SAPAS score of less than 3 would allow for the detection of a specificity of 0.85 with 95% confidence intervals of 0.70–0.94. Based on these estimates, we randomly selected 110 individuals for the study. There was no statistically significant difference in either the age or gender of the 110 people randomly selected for the study, compared with those not selected from the eligible pool.

### Measures

#### Screening measure

The SAPAS consists of eight questions, corresponding to a descriptive statement about the person. Each question is scored 0 (No)/1 (Yes), except for question 3 which is inversely scored 1 (No)/0 (Yes). The scores on the eight items are added together to produce a total score ranging between 0 and 8. The full text of the questions can be found in the original SAPAS validation study by Moran et al. (Moran et al., 2003).

#### Reference standard

The SCID‐II (First, Spitzer, Gibbon, & Williams, 1995a) was selected as the reference standard in this study. The SCID‐II is a 119‐item semi‐structured face‐to‐face interview. Each item is scored as 1 (absent), 2 (subthreshold) or 3 (threshold). Questions may necessitate further exploration by the interviewer in order to score a particular item. If a threshold is reached on a sufficient number of items, the category of PD is deemed to be present. The SCID–II was designed to generate DSM–III–R diagnoses; however, by eliminating items for passive–aggressive and depressive PDs, it can be used to generate DSM–IV PD diagnoses.

#### Procedure

South East London Community Health study participants were recruited between 2008 and 2010. Baseline screening with the SAPAS was performed by interviewers as part of a face‐to‐face interview battery. SCID‐II interviews were conducted in 2012 by a trained clinical interviewer (M. F.) experienced in psychiatric diagnosis, who was blind to the participant's baseline SAPAS score. Participants were paid £15 for giving up their time to participate in the study. Written informed consent was obtained from each participant prior to interviewing.

### Data analyses

The aim of the statistical analysis was to evaluate the ability of SAPAS to discriminate between patients with and without DSM–IV Personality Disorders (SCID). Towards this end, we used a receiver operating characteristic (ROC) analysis to assess the performance of the SAPAS and to identify an appropriate general population cut‐off score on the SAPAS for predicting a diagnosis of any PD (present or absent) on the SCID‐II. An ROC curve was obtained by plotting pairs of true positives (sensitivity) against false positives (1—specificity) for all possible cut‐off scores on the SAPAS. The area under the curve (AUC) was calculated to provide an estimate of the screen's discriminatory performance. The sensitivity and specificity of the SAPAS at different cut‐off scores was assessed using a sensitivity–specificity plot. The internal consistency of the SAPAS was assessed by calculating Cronbach's alpha on the total score after omitting each item and also overall. All analyses were performed using SAS v9.3 (SAS Institute, Cary, NC, USA). All *p*‐values given are two sided; the level of significance was set to 0.05.

## Results

All 110 participants who were randomly selected and invited to participate in the study agreed to take part. The mean age of the sample was 42.8 years (SD = 15.7). Sixty‐five (59%) were female, 72 (65%) were White, 21 (19%) were Black‐Caribbean or Black African and the remainder (*n* = 17; 15%) were from a range of other ethnic groups. A total of 35 out of 110 persons received a SCID‐II diagnosis of PD, giving an overall prevalence of 32%. The mean number of PD diagnoses among those with any PD was 1.4 (SD = 0.65). The mean time in days between SAPAS screening and SCID‐II interview was 948 days (SD = 202.6 days, range 461 to 1327 days).

**Figure 1:**
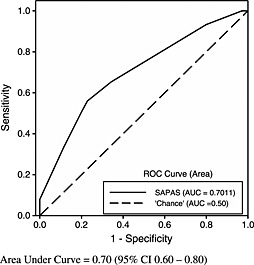
Receiver operating characteristic (ROC) curve for Standardised Assessment of Personality – Abbreviated Scale (SAPAS) as a screen for any Structured Clinical Interviews for DSM‐IV Personality Disorder (SCID‐II)

Figure 1 displays the ROC curve for the SAPAS as a screen for a SCID‐II criterion diagnosis of any PD in the sample. The ROC curve for a well‐performing test deviates significantly from the 45° reference line (AUC = 0.5), where no discrimination exists, and approaches the ‘ideal test point’ (AUC = 1), where sensitivity and specificity both equal 100% and the false positive rate is 0% (Kongerslev et al., 2012; Kraemer, 1992). In our sample, the ROC curve for the sample was statistically highly significantly different (*p* < 0.001) from the 45° reference line. The AUC was 0.70 (95% CI = 0.60 to 0.80), indicating moderate overall discriminatory accuracy, taking all possible cut scores into account.

**Figure 2:**
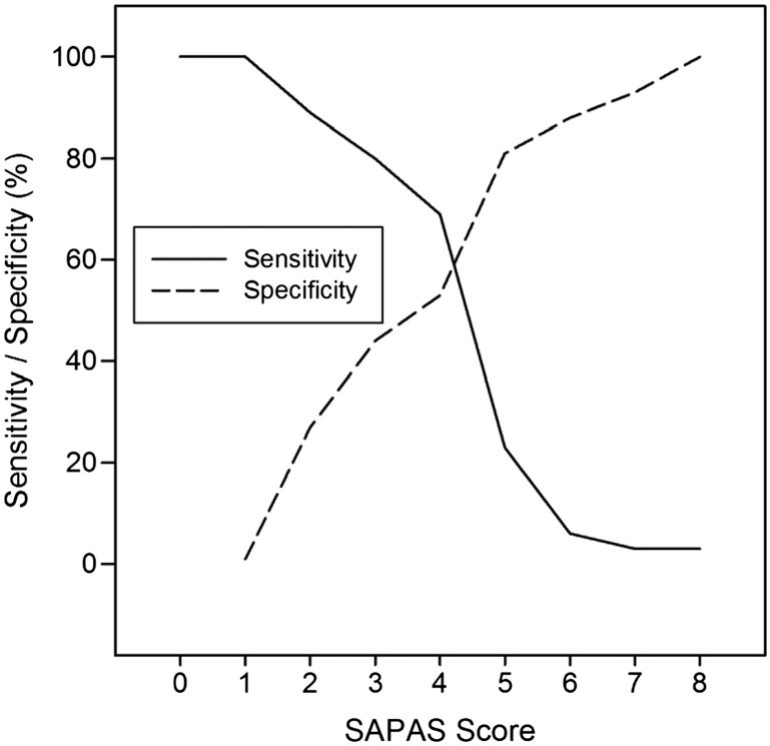
Sensitivity–specificity plot relating Structured Clinical Interviews for DSM‐IV Personality Disorder (SCID‐II) positive diagnosis to total score on the Standardised Assessment of Personality – Abbreviated Scale (SAPAS)

The performance of the SAPAS at a range of cut‐off scores is displayed in Table 1. These data, together with the sensitivity–specificity plot (Figure 2) revealed that the optimal SAPAS cut‐off score for a SCID‐II‐based diagnosis of PD was 4. A cut‐off score of 4 had the best balance of sensitivity and specificity and correctly classified 58% of individuals.

**Table 1:** Sensitivity, specificity and power to predict personality disorder at different cut‐off scores of the Standardised Assessment of Personality – Abbreviated Scale

Cut‐off score	Sensitivity	Specificity	Positive predictive value	Negative predictive value	Correctly classified (%)
2 or more	0.89	0.27	0.36	0.83	46
3 or more	0.80	0.44	0.40	0.83	55
4 or more	0.69	0.53	0.41	0.78	58
5 or more	0.23	0.81	0.36	0.69	63

Table 2 shows the alpha coefficients of each item from the SAPAS. The alpha coefficient for the total SAPAS score was 0.51, with ‘Generally a perfectionist’ being the item that was least consistent with the remaining items.

**Table 2:** The internal consistency of the Standardised Assessment of Personality – Abbreviated Scale

Item	Alpha coefficient if item omitted
Difficulty making and keeping friends	0.47
Usually a loner	0.42
Trusting others	0.46
Normally loses temper easily	0.41
Normally impulsive	0.45
Normally a worrier	0.48
Depend on others a lot	0.47
Generally a perfectionist	0.58

The alpha coefficient for the total score is 0.51

## Discussion

To our knowledge, this is the first attempt at validating a PD screening tool in a general population sample. The study provides evidence of the performance of the SAPAS as a screening measure for PD in the general population. The ROC analysis showed that the SAPAS has moderate discriminatory accuracy (AUC = 0.7) and a cut point of 4 provided the optimal balance between sensitivity and specificity. However, this cut point correctly classified the presence of PD in only 58% of participants. In addition, the internal consistency of the SAPAS in the general population was low (α = 0.51). Regarding this latter finding, it is important to stress that the internal consistency obtained in this study is comparable with that obtained in other samples (Germans, Van Heck, Moran, & Hodiamont, 2008). Moreover, alpha values are dependent on the number of scale items and the dimensionality of the underlying construct being measured (Kongerslev et al., 2012). The SAPAS is screening for a heterogeneous and multidimensional construct (the broad category of PD), and we would therefore not anticipate a high level of internal consistency.

The findings should be considered in the light of a number of limitations. The long time lag between SAPAS screen and SCID‐II interview, from a minimum of 15 months to a maximum of over 3 years, means it is possible that real change in personality function occurred in some participants between initial SAPAS screening and subsequent SCID‐II interview; this would have led to substantial underestimation of the predictive power of the SAPAS. In other words, the apparent failure of the SAPAS to correctly identify a sizeable proportion of people with PD in this study may partially reflect underlying changes in the PD status of the study participants over time. Although the SCID‐II is a widely used and well‐established instrument for diagnosing PD, our choice of this measure as the ‘gold standard' against which we validated the SAPAS could be questioned, particularly as the test–retest reliability of the instrument is less than optimal in non‐clinical samples (First et al., 1995b).

The study findings differ in some respects from previous validation studies of the SAPAS. The original study by Moran et al. (2003) validated the SAPAS in adult psychiatric patients recruited in outpatients, day‐patients and inpatient units in London, UK, and found that a cut point of 3 or 4 correctly classified PD in over 80% of participants, with a cut point of 3 offering the best balance of sensitivity (0.94) and specificity (0.85). Further studies in samples of incarcerated adolescent boys (Kongerslev et al., 2012) and probationers (Pluck et al., 2012) found a cut point of 3 correctly identified PD in 86% and 78% of participants respectively. Two studies using a self‐report version of the instrument, in psychiatric outpatients and inpatients with substance dependence respectively, both found the optimal cut point to be 4 (Germans et al., 2008; Gonzalez, 2014).

Compared with these previous studies, our study found that the SAPAS had a diminished predictive power in a general population sample. To date, the SAPAS has been applied primarily to clinical samples, and our findings suggest this is where its continued use is best justified. Our findings concord with Morse and Pilkonis' investigation of the validity of three PD screening measures (Inventory of Interpersonal Problems Personality Disorder Scale; Temperament and Character Inventory Self‐Directedness Scale; and Iowa Personality Disorder Screen) in psychiatric and non‐psychiatric samples (Morse & Pilkonis, 2007), in which all three screening measures were shown to be more effective in a psychiatric sample than in a non‐psychiatric sample—in the non‐psychiatric sample, none of the three screens had a statistically significant AUC, and diagnostic efficiencies (i.e. percentage correctly classified) were in the range of 50–60%.

The difficulties of exporting screening measures, developed in clinical samples, to non‐clinical samples is likely a result of spectrum bias (Ransohoff & Feinstein, 1978). The causes of spectrum bias may include the following: (1) the lower prevalence of the disease in the general population compared with clinical settings; (2) the differences between disease‐positive individuals in the general community setting and those in a clinical setting; and (3) the differences between disease‐negative individuals in the general community setting and those in a clinical setting. In other words, apart from the difference in base rates of the disease between the general population and clinical settings, the phenomenon under investigation may also differ qualitatively, much like the controversy about whether the ‘nature’ of depression—not only its severity—is different in primary care settings than in psychiatric settings (Klinkman, 2003; Vuorilehto, Melartin, Rytsala, & Isometsa, 2007). We detected a difference in the severity of personality disturbance between individuals in our community study (where the mean number of PD diagnoses was 1.4) compared with individuals in a clinical population used in the original validation study (where the mean number of PD diagnoses was 2.1) (Moran et al., 2003). In Morse and Pilkonis' study (Morse & Pilkonis, 2007), the test–retest reliability of screeners was lower in non‐psychiatric samples, perhaps indicating that people with PD in non‐psychiatric samples have a lesser fixity and pervasiveness of personality pathology compared with those in psychiatric samples.

The challenge for a screening tool to identify cases from non‐cases of PD should also be considered in the context of the number of PD diagnoses represented in the DSM—the 10 different specific single PD diagnosis and the possibility of a mixed or comorbid presentation (Morse & Pilkonis, 2007). A screening tool that endeavours to screen for the presence or absence of any PD has a much higher aim than one screening for a specific single PD.

Since its development in 2003, the SAPAS has been widely applied in research and clinical practice. Its key advantages over other PD measures are as follows: it is a very rapid screen (taking less than 2 min to complete); does not require training; is simple to use; and is acceptable to respondents. Moreover, it has good predictive utility for a range of clinical settings—for example, as a predictor of response to antidepressant treatment (Gorwood et al., 2010), in predicting dropout from specialist services (Crawford et al., 2009), and as a marker of problems in addiction populations (Hesse et al., 2008). To our knowledge, no other PD screen has had its performance tested in the general population, and our findings suggest that the SAPAS is best used in clinical samples. Nevertheless, the instrument may still have utility for capturing personality dysfunction in large studies of general populations (Fok et al., 2014a; Solmi, Hatch, Hotopf, Treasure, & Micali, 2014).

## Declaration of interest

The views expressed are those of the author(s) and not necessarily those of the NHS, the NIHR or the Department of Health. On behalf of all authors, the corresponding author states that there is no conflict of interest.
